# Antibiotics and Antimicrobials for Treatment of the Oral Microbiota: Myths and Facts in Research and Clinical Practice

**DOI:** 10.3390/antibiotics9020095

**Published:** 2020-02-22

**Authors:** Gaetano Isola

**Affiliations:** Unit of Oral Surgery and Periodontology, Department of General Surgery and Surgical-Medical Specialties, School of Dentistry, University of Catania, Via S. Sofia 78, 95124 Catania, Italy; gaetano.isola@unict.it

**Keywords:** periodontitis, antibiotics, periodontal therapy, resistance, antimicrobials, clinical trial

## Abstract

In the dental field, the most common oral diseases include periodontitis, apical periodontitis, abscesses, phlegmons and pulpits, all of which are determined by the same aetiological factor, bacterial infections. For these reasons, it is important to choose the right approach through a target antibiotic therapy against oral bacteria. More specifically, during periodontitis, antibiotics are used, often in association with periodontal debridement, to reduce disease-associated periodontopathogens. However, international guidelines are not unanimous in recommending the use of local and/or systemic antimicrobials to reduce infection by oral bacteria, especially in cases in which there is a danger of spreading systemic infection such as cellulitis, diffuse swelling, and abscesses. The lack of consensus is mainly due to the side effects of antibiotic therapy in dentistry, maybe due to recent scientific evidence regarding the development of bacterial resistance to antibiotics. Therefore, the purpose of this editorial is to analyze the therapeutic effects of antibiotics against the main forms of oral and periodontal diseases, and whether there is a significant clinical benefit, especially in the long term, of antimicrobial therapies in dentistry. The most recent evidence regarding antimicrobial agents will also be discussed.

## Editorial

Worldwide, periodontitis is one of the most common chronic inflammatory diseases of the soft and hard tissues of the tooth [1]. More specifically, in Europe each year, approximately 40% of the population suffers from mild to advanced forms of periodontal disease that requires appropriate treatment [2,3]. If periodontitis remains untreated, it can cause a significant degradation of periodontal tissues over time, which, in turn, causes apical migration of the junctional epithelium, and in the final stages, tooth loss [4,5,6]. If periodontal disease is not adequately treated, it can also lead to a significant reduction in chewing and aesthetic functions, which can be accompanied by impairment of social life and relationships in the most extensive cases [7,8,9].

Chronic inflammation associated with periodontitis not only affects, on a local level, the integrity and functions of the periodontium and its related tissues, but also leads to a marked increase in inflammation, both locally and systemically [10].

According to this theory, several large-scale epidemiological studies have shown a close relationship between the presence of periodontal disease and the early onset of some systemic diseases, including diabetes mellitus [11], metabolic and pulmonary diseases, rheumatoid arthritis [12,13], stroke and cardiovascular diseases [14,15]. During periodontitis, there is a chronic systemic inflammatory reaction and transmigration of oral bacteria (i.e., periodontal pathogens), which ascend from the gingival pocket to the systemic vascular pathway, causing a close interaction with the vascular endothelial cells that increases the risk of endothelial dysfunction.

On the other hand, the etiology of periodontitis is closely linked to the development of a process known as “dysbiosis” in which the saprophytic bacteria of the oral cavity are progressively reduced at the expense of the periodontal pathogenic bacteria of the oral biofilm. These are mostly Gram-negative, and are responsible for the beginning of a highly specific and sometimes aggressive proinflammatory response [16,17,18]. Therefore, reduction of bacterial biofilms, especially those caused by pathogenic bacteria including *Porphyromonas gingivalis*, *Treponema denticola* and *Tannerella forsythia*, must be the goal of any type of surgical or non-surgical periodontal therapy. Accordingly, in the periodontal literature, the treatment of periodontitis first includes the mechanical non-surgical removal of the bacterial biofilm accompanied by home oral hygiene, which must be followed by the patient together with a recurrent professional therapy called "periodontal maintenance therapy" [19,20,21].

The majority of periodontal interventional studies have shown a statistically significant microbiological benefit from the use of adjuvant administration of systemic antibiotics in combination with short-term non-surgical mechanical therapy, although their long-term clinical relevance is still debated [22]. The real effectiveness of antibiotic therapy, both in systemic form and in localized form, has been questioned in light of the risk of development of microbial resistance and also the negative influence of this therapy on the human microbiome, especially with regard to the empirical and extensive use of antibiotics in the treatment or prophylaxis of oral infections [23,24,25,26,27,28]. The appropriateness of antibiotics in dentistry and in periodontology must, in the light of recent scientific evidence, certainly be reassessed critically, especially in light of the additional long-term benefit and of all possible adverse drug reactions that may arise for the patient [29,30,31,32,33]. In this context, there is also an absence of concrete guidelines to advise the clinician in administration of additional systemic antibiotics for treatment of oral and periodontal diseases [34,35,36,37].

The purpose of this report and Special Issue is to provide the clinician and dental researcher with additional decision support and evidence on the effects of administration of systemic and/or topical antibiotics effective in oral and periodontal therapies.

Specifically, topics analyzed include the most recent evidence-based analyses of the appropriateness and benefit of antibiotics as an adjunct therapy to the mechanical removal of biofilm for treatment of various oral pathologies. Moreover, the topic of whether there is evidence in the indication of antibiotics on the basis of the severity of the oral disease being treated is addressed, as well as the comorbidities associated with antibiotic therapy in dentistry.
